# Exploring Physicians’ Perspectives on the Introduction of Complementary Foods to Infants and Toddlers

**DOI:** 10.3390/nu13103559

**Published:** 2021-10-12

**Authors:** Łukasz Dembiński, Aleksandra Banaszkiewicz, Katarzyna Dereń, Aleksandra Pituch-Zdanowska, Teresa Jackowska, Jarosław Walkowiak, Artur Mazur

**Affiliations:** 1 Department of Pediatric Gastroenterology and Nutrition, Medical University of Warsaw, 02-091 Warsaw, Poland; aleksandra.banaszkiewicz@wum.edu.pl (A.B.); aleksandra.pituch@wum.edu.pl (A.P.-Z.); 2 College of Medical Sciences, University of Rzeszow, 35-310 Rzeszów, Poland; kderen@ur.edu.pl (K.D.); drmazur@poczta.onet.pl (A.M.); 3 Department of Pediatrics, Center of Postgraduate Medical Education, 01-813 Warsaw, Poland; tjackowska@cmkp.edu.pl; 4 Department of Pediatric Gastroenterology and Metabolic Diseases, Poznan University of Medical Sciences, 60-572 Poznan, Poland; jarwalk@ump.edu.pl

**Keywords:** complementary feeding, family counseling, nutrition, pediatrics, weaning

## Abstract

Complementary feeding is the subject of many recommendations regarding the benefits of its use, illustrating its crucial impact on further health. However, it still poses a significant problem for caregivers, and thus for doctors. This survey focused on nutritional problems faced by the parents of infants and toddlers, as well as how physicians deal with these problems. Based on the responses from 303 doctors, it was determined that the time and sequence of introducing complementary foods raise the greatest doubts in parents. This study also found that at least one-third of pediatricians experience difficulties in providing effective nutritional counseling. Increasing the nutritional awareness of physicians can allow them to provide more appropriate support to parents.

## 1. Introduction

Despite existing guidelines and recommendations from scientific societies, feeding infants and toddlers is one of the most common subjects of debate amongst pediatricians, dieticians, and obviously, parents [1,2]. The weaning from exclusive breastfeeding and the introduction of complementary foods are particularly emotive topics.

Complementary foods should be introduced when exclusive (breast- or formula-) milk feeding becomes insufficient for growing infants to meet their nutritional requirements. However, the term “insufficient” is not precisely defined. The time of introducing solid foods and liquids, other than breast- or formula milk, has been established as not earlier than 17 weeks, which means the beginning of the fifth month of life and no later than 26 weeks, being the beginning of the seventh month. Any relationship with timing of introduction of complementary feeding and the risk of development of some chronic diseases like allergies, asthma or celiac disease, according to available data is not conclusive. However, it has been claimed that there is no need to delay the introduction of allergenic foods as a form of prevention therapy [3,4,5]. Moreover, complementary feeding should be adequate in quantity, quality, textures, and variety, and should be tailored to the child’s psychomotor skills, health condition, and family eating habits. Additionally, there is growing evidence that salt, sugar (especially found in juices or sugar-sweetened beverages), and many other diet ingredients may negatively impact a child’s future health [5,6]. 

In daily practice, a doctor’s consultation time is limited, and quite often, does not allow for the discussion of all details of a child’s feeding. Therefore, physicians choose to discuss only a limited range of issues with parents; namely, those they feel are most important. However, it is unclear whether the doctor’s choices and the parent’s needs are identical. 

This study aimed to assess the most common and urgent questions and issues asked by parents to their pediatricians regarding feeding infants and toddlers, and what is the pediatricians’ perception of their role in this issue.

## 2. Materials and Methods

This study was initially intended to be conducted based on focus groups of pediatricians and parents. Unfortunately, the COVID-19 pandemic forced the modification of these assumptions and the creation of an online questionnaire. The online survey was designed using the Google Forms platform to assess physicians’ perspectives and attitudes on introducing complementary foods to infants and toddlers. The link to the survey was sent by mail to the registered in the electronic database members of the Polish Society of Pediatrics (PTP, Polskie Towarzystwo Pediatryczne), and participants were recruited in May–June 2021. In addition to basic questions about each physicians’ age and experience, the survey contained 13 multiple-choice questions focused on nutritional problems faced by the parents of infants and toddlers, and how the physicians were dealing with them (Supplementary Table S1). In addition, five of these questions allowed the participants to share their own opinions; for example, perspectives were sought on the impact of infant nutrition on health in later life and the differences between commercial baby foods and home-prepared ones. Statistical analysis was performed using the Statistica software, version 13.1 (TIBCO Software Inc., Palo Alto, CA, USA).

## 3. Results

The invitation to complete the survey was sent to 1977 pediatricians. 303 (15.3%) questionnaires containing complete answers were included in the statistical analysis. Among the participants, 80.9% (245/303) were women, and 47.2% (143/303) of the physicians had over ten years of professional experience, 55.9% (80/143) of which exceeded 20 years of experience. 81.2% (246/303) of the doctors primarily specialized in pediatrics, and the remaining 57 identified themselves as family doctors or general practitioners. Among the participants, 66.6% (202/303) primarily worked at a hospital, while the remaining 101 were working at outpatient clinics.

Of the participants’ knowledge on complementary food introduction, 49.8% (151/303), 32.7% (99/303), and 11.2% (34/303) were derived from national and international guidelines, training conferences, or acquired during studies or specialist training, respectively. More than half of the participants (58.7%, 178/303) considered their knowledge in this area to be sufficient and up-to-date. Among the participants, 67.0% (203/303) believed that complementary feeding practices were based on scientific research. Virtually all participants (99.0%, 300/303) agreed that feeding practices used in infants and toddlers could influence health in adulthood. The participants provided examples of these influences, such as the development of correct eating habits and reducing the risk of obesity and diet-related diseases.

In their practice, 62.7% (190/303) of physicians received questions about introducing complementary food from every third parent, and 86.8% (263/303) asked questions about this issue to every third patient. According to doctors’ perception, 75.6% (229/303) of parents expected to receive scientific advice, while only 48.5% (147/303) expected emotional support. The majority (71.0%, 215/303) of participants thought that the parents perceived them as an authority in the field of nutrition, although as many as 32.7% (99/303) experienced some kind of difficulties in convincing parents to follow their advice.

According to the participants, 93.4% (283/303), 51.5% (156/303), 45.9% (139/303), and 32.7% (99/303) of parents gained their knowledge about the introduction of complementary foods from the Internet, other parents, family, and parenting guides, respectively. Doctors were the primary source of knowledge for only 18.5% (56/303) of parents.

Parents most often asked doctors about the age at which complementary foods should be introduced, the order in which the foods should be introduced, and about adequate drinks for children. According to physicians, the biggest problem for parents was understanding what a child could drink and the order in which complementary products should be introduced. Detailed data are presented in Table 1.

During family counseling, 51.8% (157/303) of doctors recommended commercial baby foods for complementary food introduction, 34.0% (103/303) recommended food prepared at home, and 14.2% (43/303) left this decision to the parents. Doctors justified their responses based on the quality, known composition, and safety of the ready-made products.

## 4. Discussion

According to our knowledge, this is the world’s first survey that explored physicians’ perspectives on complementary feeding in infants and toddlers. So far, only studies on physicians’ attitudes and practices towards breastfeeding have been published [7,8,9]. Although the issues regarding the introduction of foods to infants and toddlers are not any less important, they have not been previously analyzed this way. We would like our study to be the first of a larger project, and we plan to continue exploring this topic in the nearest future.

In our survey, only slightly more than 15% of the respondents answered all the questions. However, this study was conducted during the COVID-19 pandemic, when much research was done electronically. This may have contributed to some “fatigue” of potential respondents with this form of surveying. We also assume that mainly those pediatricians who deal with nutrition counseling on a daily basis decided to participate in this survey. Women were dominant among the responding physicians, which is consistent with the gender distribution among the members of the PTP (over 70% of women).

Based on our results, it appears that the primary concerns for caregivers, from the doctor’s point of view, include the age and the order at which foods should be introduced, and the appropriate drinks for children. Previous studies show that, although 80% of caregivers were familiar with current feeding recommendations, only a third followed them precisely [10,11,12]. These studies corroborated our results, which demonstrated that the most common deviations were related to the age at which specific foods were introduced and the early introduction of sweetened beverages [13,14,15]. Proper medical guidance can potentially reduce these faults, the avoidance of which is especially important in this era of overweight and obesity epidemics [16].

The results regarding the choice between commercial baby foods and home-cooked meals were slightly unexpected. Only a third of the participants recommended home-cooked meals, and more than half recommended commercial baby foods. Complementary foods for infants and toddlers can be homemade or bought as ready-to-eat commercial products. The nutrient composition of commercial products is regulated by the European Commission Directive, while the preparation of homemade complementary meals is the responsibility of caregivers [17,18]. Research in Germany has shown that infants that were fed with commercial meals at 12 months of age received a wider variety of vegetables than those fed with homemade meals, and in general, there has been a lack of specific, consistent international guidelines for such meals [18]. Carrots were most commonly used vegetable in homemade and commercial meals, whereas other vegetables were used far less frequently. Personal authors’ experience leads us to affirm that the same happens in Poland. In both types of meals, poultry and beef were the most often used, whereas fish meals were rarely offered [19]. In contrast, studies in Canada have shown that providing homemade complementary foods was associated with increased dietary variation during the first year of life [20]. The newest study from Spain showed that homemade meals contained a significantly greater number of different vegetables than commercial meals, and both of them were the most abundant in carrot. In relation to fruits, the reverse was found, the variety was greater in commercial meals, but in both a banana was the most frequently present in fruit purees. In both types of complementary meals, there was a predominance of meat and a scarcity of legumes and fish oil [21]. Bernal et al. have shown that homemade meals were significantly lower in energy and had higher protein and fiber content than commercial meals [21]. Different results in different contexts and countries outline the impact that a diverse culture can have in this aspect, thus emphasizing the difficulties in implementing unique guidelines, applicable globally. The complementary food period is a process by which the child is progressively led to the food culture of his/her own family, thus indications valid for some parents can be useless or not applicable to other families, even within the same country. Again, it should be emphasized that research shows that there are no significant differences between commercial and homemade complementary meals, except for higher sodium content in some commercial savory meals for older infants, lower fat content in commercial non-fruit-based or cereal-fruit meals, and added sugar content in some commercial dairy-fruit meal [18]. Although, we cannot estimate the content of these additives in every homemade meal due to the huge variety of recipes and family habits. As there are no dieticians in outpatient clinics and dieticians are present only in some selected hospitals, it becomes the duty of the doctors to counsel these aspects of complementary foods. It is necessary that they should guide parents in the right direction pointing crucial aspects of choosing commercial or homemade foods for infants, taking account of parents’ degree of knowledge about food safety and their cooking and food preparation skills.

Almost half of the participants of our survey declared that they obtained their knowledge directly from national and international guidelines, and more than half considered their knowledge to be up-to-date and sufficient. Furthermore, more than two-thirds of the pediatricians in our study believed that weaning and complementary foods introduction practices were based on scientific evidence. In addition, almost all of them were convinced that nutrition in the first years of life significantly impacted health in adulthood, including the reduction of the risk of diet-related diseases. These are very encouraging results. However, some studies show that physicians often misunderstand the nutritional guidelines, thus it is worth clarifying and discussing these guidelines, for example, during training meetings and workshops [22,23,24]. As a matter of fact, knowledge on the influence of nutrition during the first years of life on later health has grown significantly in recent years, both among doctors and the general population [25,26]. Nonetheless, studies from various countries confirm that physicians show little knowledge of clinical nutrition, and even though they perceive nutrition counseling as a priority, they lack the confidence and knowledge to effectively provide adequate nutritional advice to their patients [27,28,29]. Furthermore, a systematic review of the literature has shown that nutrition is insufficiently considered and dealt with in medical education, regardless of the country or year of medical education [28]. Despite the critical importance of nutrition for a healthy lifestyle, the education received by medical graduates does not enable them to provide patients with high-quality and effective nutritional care [30]. Therefore, it is essential to encourage doctors to constantly update their knowledge and nutrition, especially during first year of life, and it should be an important part in the continuing medical education. 

There is a strong discrepancy between who pediatricians consider think they are for parents, that is, as an authoritative source of information, and the statement by the pediatricians themselves that in fact parents rely on the web for their choices. [31,32]. According to our results, parents obtain their knowledge about children’s nutrition mainly from the Internet, and doctors remain the primary source of knowledge for only 18.5% of caregivers. Unfortunately, this is consistent with the results of other studies, which showcase the dominant role of the Internet in obtaining health information by parents, and the poor influence of pediatric counseling, resulting in no changes to the parental behaviors regarding the complementary feeding process [33,34]. Therefore, efforts should be made to strengthen the role of pediatricians as a reliable source of information on the nutrition of infants and toddlers. Parents need practical tips, which must be based on formal recommendations, so that some well-prepared educational materials with practical schemes, recipes, information about properly preparing homemade meals for infants, should be always provided to patients during visits. Thus, doctors will have specific practical tools to improve the quality of the visit regarding nutrition aspects of an infant.

Most of our recruited participants (76%) believed that caregivers ask for scientific advice or at least scientific information, while less than half indicated that emotional support was a caregiver’s primary concern. Research on parental expectations has shown that both elements are closely connected [35]. Parents attending well-child care visits need the reassurance about their child’s proper development, but seek also the opportunity to clarify their doubts [35,36].

This is the world’s first study that assessed physicians’ perspectives on introducing complementary foods to children, which is the main advantage of the study. The main limitation of this study is the size of the recruited group. The small number of participants could be due to the fact that many pediatricians do not consider complementary feeding an important topic or that many of them have been tired of responding and interacting with online tools due to their abundant use during the pandemic. Asch et al. in their study analyzed over 400 medical surveys to assess response rates to mail surveys published in medical journals. They found that higher response rates were associated with surveys of non-physicians; physicians had the lowest mean response rate among all groups examined. In addition, response rates were lower in surveys if the surveys were anonymous, and were higher if they used any written reminder with an instrument or any telephone reminder [37]. A second limitation is that the study includes only physicians and only from one single country, and consequently, the results cannot be directly extrapolated for other European countries. However, it should be noted that the system of education and specialist training is quite similar across Europe, and thus should not significantly affect the results. 

This study is a basis for verifying the possibility of using an online survey to learn about the attitudes and perspectives of health professionals in different fields of daily practice. As soon as the pandemic allows, focus groups with the same questions will be prepared to verify whether the results are similar in “live” mode. We hope that this questionnaire-based study will help physicians not only identify parental expectations but also how to cope with feeding counseling.

## 5. Conclusions

According to the results of this online survey on pediatricians’ perspectives, the main parental challenges in complementary feeding are the age at which complementary foods are introduced, the order in which complementary foods should be introduced, and the appropriate choice of beverages. 

Promotion of continued nutritional education, considering a complementary feeding process is required, and should be warranted for doctors as an essential health practice.

## Figures and Tables

**Table 1:** Questions parents ask their doctors and the lacks in parental knowledge as assessed by pediatricians.

	Parents’ Doubts	Lacks in Parents’ Knowledge
number and volume of meals	53.5% (162/303)	44.2% (134/303)
age (i.e., month) of introduction of complementary foods	72.9% (221/303)	47.2% (143/303)
order in which complementary foods should be introduced	67.7% (205/303)	54.5% (165/303)
time of complete weaning	36.3% (110/303)	28.7% (87/303)
method of introducing new foods (e.g., baby-led weaning, traditional weaning practices)	36.0% (109/303)	31.0% (94/303)
consistency of the introduced foods	21.5% (65/303)	24.1% (73/303)
differences between ready-made and home-made foods	22.8% (69/303)	20.5% (62/303)
children’s drinks (e.g., juices vs. water)	61.1% (185/303)	64.7% (196/303)

## Data Availability

Original data are available from the first author upon rational request.

## Supplementary Materials

The following are available online at https://www.mdpi.com/article/10.3390/nu13103559/s1, Table S1: Questions form survey exploring physicians’ perspectives on the introduction of complementary foods to infants and toddlers.
