# Supplementary material for: Exploring Physicians’ Perspectives on the Introduction of Complementary Foods to Infants and Toddlers
Source: Nutrients. 2021 Oct 12;13(10):3559. doi: 10.3390/nu13103559 (PMC8541593; doi:10.3390/nu13103559)
Supplement: Supplementary file 1 [file nutrients-13-03559-s001.zip › nutrients-1378402-supplementary.pdf]

Supplementary Table S1. Questions form survey exploring physicians' perspectives on the introduction of complementary foods to infants and toddlers.

Questions	Possible Answers
How often do parents ask you about infant nutrition?	never<br>sporadically (e.g., single questions per month)<br>often (e.g., every third or fourth parent)
How often do you ask parents about infant nutrition?	never<br>sporadically (e.g., single questions per month)<br>often (e.g., every third or fourth parent
The most common questions asked by parents about weaning are (multiple choice):	number and volume of meals<br>age (e.g., month) of introducing complementary foods<br>order in which complementary foods should be introduced<br>time of weaning<br>way of feeding (e.g., BLW)<br>consistency of the introduced foods<br>differences between ready-made and homemade foods<br>children's drinks (e.g., juices or water)<br>other (please specify)
In your opinion, parents lack knowledge about (multiple choice):	number and volume of meals<br>age (e.g., month) of introducing complementary foods<br>order in which complementary foods should be introduced<br>time of weaning<br>way of feeding (e.g., BLW)<br>consistency of the introduced foods<br>differences between ready-made and homemade foods<br>children's drinks (e.g., juices or water)<br>other (please specify)
Where do parents most often get knowledge about infant nutrition, in your opinion? (multiple choice)	from the doctor<br>from a nurse or midwife<br>from other parents<br>from family members<br>from the Internet<br>from parenting guides
Do you find it difficult to convince families to follow your advice on feeding babies during weaning?	no<br>yes
Do you think parents consider you an authority on infant nutrition?	no<br>yes
When it comes to infant nutrition, do you think parents expect from you mainly:	scientific knowledge<br>emotional support
In your opinion, weaning practices:	have a solid scientific basis<br>are mainly based on empirical knowledge
Do you think that nutrition during the first year of life can affect health in adulthood?	no<br>yes (please specify)

Where do you mainly get your knowledge of infant nutrition from?	from the knowledge gained during studies and specialization<br>from previous experience<br>from scientific and training conferences<br>from national and international guidelines
Do you consider your knowledge of infant nutrition sufficient and up-to-date?	no<br>yes
At weaning, usually do you suggest homemade food or commercial baby food?	homemade food<br>ready-to-eat commercial products commercial food<br>(please specify why)
